# *Pistacia lentiscus*: Phytochemistry and Antidiabetic Properties

**DOI:** 10.3390/nu16111638

**Published:** 2024-05-27

**Authors:** Sonia Floris, Amalia Di Petrillo, Francesca Pintus, Giovanna Lucia Delogu

**Affiliations:** 1 Department of Life and Environmental Sciences, University of Cagliari, 09042 Monserrato, Italy; s.floris@unica.it (S.F.); delogug@unica.it (G.L.D.); 2 Department of Medical Sciences and Public Health, University of Cagliari, 09042 Monserrato, Italy; amalia.dip@unica.it

**Keywords:** *Pistacia lentiscus*, Chios mastic gum, diabetes, therapeutic target, hypoglycemic effect, hypolipidemic effect

## Abstract

*Pistacia lentiscus* L. (*P. lentiscus*) is an evergreen shrub (Anacardiaceae family) primarily found in the Mediterranean region. The plant has been thoroughly characterized, resulting in a high concentration of bioactive compounds as flavonoids and phenolics. Moreover, *P. lentiscus* was revealed to possess a great nutritional and industrial importance because of its variety of biological activities, including antibacterial, anti-inflammatory, anti-atherogenic and antioxidant properties. Many of its beneficial health properties and applications date back to antiquity, and the European Medicines Agency officially acknowledged it as an herbal medicinal product. Indeed, it is widely employed in conventional medicine to treat several diseases, including type 2 diabetes (T2D). On this basis, this review aims to summarize and describe the chemical composition of different parts of the plant and highlight the potential of *P. lentiscus*, focusing on its antidiabetic activities. The plant kingdom is drawing increasing attention because of its complexity of natural molecules in the research of novel bioactive compounds for drug development. In this context, *P. lentiscus* demonstrated several in vitro and in vivo antidiabetic effects, acting upon many therapeutic T2D targets. Therefore, the information available in this review highlighted the multitarget effects of *P. lentiscus* and its great potential in T2D treatment.

## 1. Introduction

*Pistacia lentiscus* L. is an evergreen shrub belonging to the Anacardiaceae family, mainly distributed in Mediterranean region. It is commonly used in traditional medicine for the treatment of several diseases, including diabetes [1,2,3]. This plant is also known as lentisk or mastic tree. The latter name derives from the presence of an aromatic resin (mastic) which can be obtained from *P. lentiscus* trunk and branches. Chios mastic gum is exclusively produced by the mastic tree grown on the Greek island of Chios, situated in the northern Aegean Sea. Many of its beneficial properties and uses had already been described in antiquity [4], and in 2015, it was recognized by the European Medicines Agency (EMA) as an herbal medicinal product with therapeutical indication for mild dyspeptic disorders and skin inflammation as well as the healing of minor wounds [5,6]. Over the last years, mastic gum has been used as a spice in the food industry, as a food ingredient, and one of the main uses is to produce a natural chewing gum. The therapeutic potential of mastic gum in the medicinal field has also been described. Anti-inflammatory action has been reported, probably being performed via the inhibition of the production of pro-inflammatory substances. Moreover, antioxidant, anti-atherogenic, anticancer and antibacterial properties have been reported [6]. Because of the resin’s chemical complexity, it is difficult to understand which bioactive compounds are responsible for these activities, even if some of them were attributed to triterpenes and volatile compounds [5]. The other parts of the plant were extensively investigated and also resulted in a high content of bioactive compounds as phenolics or flavonoids, showing several biological activities such as antioxidant, antimicrobial, hepatoprotective and antimutagenic activities [3,7].

In this context, the present review aims to summarize and focus on the antidiabetic activity of *Pistacia lentiscus*, highlighting the great potential of this plant for diabetes treatment.

Diabetes mellitus (DM) is a progressive metabolic disease characterized by abnormal blood glucose levels. Diabetes is becoming more common worldwide as a result of unhealthy lifestyle choices, and by 2030, there will be 578 million cases of the disease [8]. Furthermore, the incidence of diabetes should be regarded as significantly higher, since 45% of individuals have undiagnosed diabetes.

Pancreatic β-cells secrete the peptide hormone insulin, which is essential for controlling blood glucose levels and energy metabolism. Numerous cellular processes, including the uptake and transport of glucose, the synthesis of glycogen, the synthesis of fatty acids and the synthesis of proteins, depend on it. Hyperglycemia eventually results from impaired normal glucose homeostasis caused by either insufficient insulin production or insulin resistance. Chronic hyperglycemia has been linked to major long-term consequences such as kidney failure, cardiovascular disease and nerve damage [9]. Diabetes can be divided into type 1 and type 2 diabetes. The most prevalent metabolic disease affecting children is type 1 diabetes (T1D), sometimes referred to as insulin-dependent diabetes [10]. Pancreatic β-cells are destroyed by the immune system through T-cell mediation in the pathophysiology of T1D. This ultimately causes the body to secrete less insulin, which causes the beginning of the disease.

Instead, type 2 diabetes (T2D) accounts for 90–95% of all instances of the disease, and it represents the most common type [11]. Insulin resistance and decreased insulin production work together to create this type of diabetes. The failure of skeletal muscle cells to absorb glucose and the increased synthesis of glucose in the liver are two examples of how the target tissues are insensitive to insulin. Obesity, physical inactivity and vitamin D insufficiency are the main risk factors linked to the development of T2D.

Insulin injections are commonly used as a form of treatment for T1D, and oral drugs in conjunction with lifestyle modifications are utilized to manage T2D [12]. Non-insulin medications obstruct the absorption of glucose, reduce hepatic gluconeogenesis and prevent the kidneys from reabsorbing glucose. α-glucosidase inhibitors, metformin and sodium-glucose co-transporter-2 inhibitors are a few examples of popular antidiabetic medications that support the abovementioned effects [13]. However, even though these medications have a significant role in lowering blood glucose levels, side effects are unavoidable.

The plant kingdom is attracting increasing interest in finding new bioactive compounds for the development of drugs, also due to the intrinsic complexity of natural compounds [14]. Over 800 plants have been reported to possess antidiabetic properties, showing less side effects when compared with synthetic drugs, and different bioactive compounds with different biological activities have been described [15,16,17,18].

*P. lentiscus* was selected as a promising source of biologically active compounds and may contribute to the development of new multitarget therapies for diabetes, showing several in vitro and in vivo activities toward different therapeutic T2D targets. Here, the chemical compositions of different parts of the plant together with *P. lentiscus* biological activities are summarized and described.

## 2. Chemical Constituents

**Figure 1:**
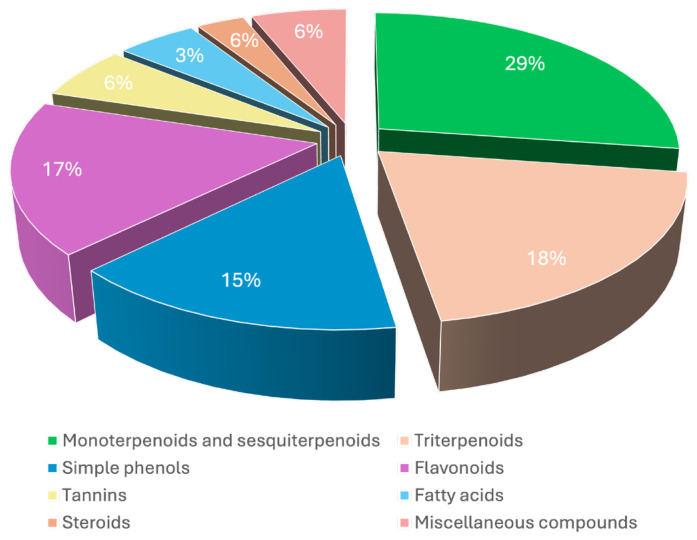
Chemical composition of *Pistacia Lentiscus*.

From the literature review, a total of 157 compounds were identified in *Pistacia lentiscus*, including essential oil constituents, monoterpenoids, sesquiterpenoids, triterpenoids, phenolic compounds (simple phenols, flavonoids, tannins), fatty acids, steroids and miscellaneous compounds. Phytochemicals were isolated from various parts of the plants, including the leaves, fruits, resin, aerial parts and barks. According to the literature, the most common methods used to identify phytochemicals from the genus Pistacia, notably from its essential oils, were high-performance liquid chromatography (HPLC) and gas chromatography-mass spectroscopy (GC-MS). These techniques allowed us to obtain qualitative and quantitative information on the biochemical constitution of *Pistacia lentiscus*. Terpenoids constitute the major chemical group, comprising approximately 47% of the total chemical constituents, followed by simple phenols, flavonoids and tannins (38%), fatty acids (3%), steroids (6%) and several miscellaneous compounds (6%) (Figure 1). Another category includes the volatile compounds included in the essential oil and mastic water, two products obtained after the distillation process of mastic gum.

Variable levels of total phenolic contents were found in all parts of *P. lentiscus*. The concentration of secondary metabolites changes significantly according to geographical origin, phenological stages, parts of the plant used, cultivation sites and extraction solvents used. A brief description of the different classes of compounds identified and their sources is shown in the following subsections.

### 2.1. Terpenoids (**1**–**75**)

Terpenoid compounds were identified in fruits and Chios mastic gum (CMG).

El Omari et al. [19] revelated that the main compounds of *P. lentiscus* essential oil extracted from fruits are α-pinene (**1**) (20.46%) and limonene (**3**, **10**) (18.26%). Consequently, both compounds were individually tested for their biological effects.

Kartalis et al. [20] described the main components of CMG being an insoluble polymer (25%) and a triterpenic fraction (67%), which is further subclassified into acidic (39%) and neutral (28%) fractions. The main components of acidic fraction are masticadienonic acid (**50**) (30%), isomasticadienonic acid (**49**) (30%), oleanonic acid (**51**) (15%) and moronic acid (**47**) (10%). The neutral fraction’s main components include butyspermol (**55**), tirucallol (**63**), oleanolic aldehyde (**57**) and betulonal (**61**). Another monoterpenic constituent of CMG is camphene (**7**), which also seems to possess promising hypolipidemic activity.

### 2.2. Phenolic Compounds (**76**–**136**)

Recent phytochemical analyses have shown that all the parts (leaf, stem, fruit and root) of *P. lentiscus* are rich in bioactive phenolic components. Among them, simple phenols were identified along with flavonoids and tannins (described in following subsections).

Several phenolic acids were detected, such as gallic acid (**85**) [1,21,22,23,24], vanillic acid (**86**) [23], several cinnamic acid derivatives such as trans-cinnamic acid (**90**) [21], p-coumaric acid (**91**) [21], caffeic acid (**92**) [21,23], ferulic acid (**93**) [25] and also tannic acid (**135**), a specific form of tannin [21].

A study on Algerian mastic tree showed that the total phenolic mass fraction in leaves as gallic acid equivalents on a dry matter basis (216.28 ± 20.62 mg/g) was significantly higher than that in stems (121.39 ± 3.35 mg/g), fruits (103.34 ± 2.32 mg/g) and roots (30.18 ± 1.29 mg/g) [2].

A study conducted by Mehenni et al. [24] asserts that the highest amounts of polyphenols obtained with an increase in the polarity of the solvent were recorded in the chloroform extracts of both leaves and fruits, followed by ethyl acetate and ethanolic extracts. Moreover, the total amounts of phenolics of leaf crude extracts were significantly (*p* < 0.01) higher than those found in fruit crude extracts (e.g., 517.512 ± 5.53 mg catechin Eq/g vs. 254.9 ± 5.04 mg catechin Eq/g detected in ethanol extract from leaves and fruits, respectively).

According to Belhachat [26] and Yosr, the total phenolic content varied from 17 mg gallic acid equivalent (GAE)/g dry matter (DM) to 955 mg GAE/g DM in the *P. lentiscus* extracts, and among the same leaf extract, changes also depended on sex (100.8 vs. 150.7 mg GAE/g DM in female and male respectively) and phenological stages (178.5 vs. 87 mg GAE/g DM in the dormancy period and late fruiting stage, respectively) [27].

#### 2.2.1. Flavonoids (**100**–**126**)

Flavonol glycosides were found to be the most abundant class of flavonoids in the leaves and fruits of *Pistacia lentiscus*. Myricetin-3-*O*-rhamnoside (**113**) [1,22] was the predominant flavonol glycoside, along with myricetin-3-*O*-glucoside (**112**) [1,2,22], quercetin-3-*O*-rhamnoside (**114**) and catechin (**102**) [2]. Myricetin derivatives account for 20% of the total amount of polyphenols in *P. lentiscus* leaves [1]. Luteolin (**100**) and apigenin (**104**), belonging to the class of flavones, were observed in high concentrations in both leaf and fruit extracts [1,24]. Mehenni et al. [24] found luteolin to be the second most abundant polyphenol in fruit. Flavonoids were identified in berries and leaves, namely, delphinidin-3-*O*-glucoside (**110**) and cyanidin-3-*O*-arabinoside (**133**) [1].

Aerial parts of fresh *P. lentiscus* collected in different areas of Sardinia showed that in the leaves, a large number of flavonoids are present, in particular members of myricetin (**105**, **113**, **114**, **120**, **121**) and quercetin (**106**, **107**, **118**, **119**) [22].

Furthermore, the oil extracted from fruits of *P. Lentiscus* (PLFO) is a rich source of phenolic compounds, with about 40 molecules identified, such as gallic acid (**85**), tyrosol (**82**), vanillic acid (**86**) and flavonoids such as kaempferol (**101**) and quercetin (**103**) [28]. The total phenolic content (TPC) and total flavonoid content (TFC) for PLFO were determined to be 25.19 ± 0.67 μg GAE/mg and 20.90 ± 4.41 μg quercetin equivalent (QE)/mg of PLFO, respectively. In the case of the unsaponifiable fraction (USM), the quantity of the TPC and TFC values was found to be 18.70 ± 2.89 μg GAE/mg and 12.5 ± 2.65 μg QE/mg of USM, respectively [28].

Furthermore, comparing different extracts from leaves and fruits, the TFC of leaf crude extracts was found to be significantly higher [24].

#### 2.2.2. Tannins (**127**–**136**)

Tannins predominate in the fruits of fresh *P. lentiscus* collected in different areas of Sardinia. Quinic acid derivatives and galloyl derivatives such as 3,5-*O*-digalloyl quinic acid (**129**), 3,4,5-tri-*O*-galloyl quinic acid (**130**), 3-galloyl quinic acid (**131**), 5-galloyl quinic acid (**132**) and 1,5-digalloyl quinic acid (**136**) were detected [1,22]. β-Glucogallin (**128**) and 5-*O*-galloylquinic acid (**132**) were instead the most abundant in leaf extract [2].

### 2.3. Fatty Acids (**137**–**144**)

Fatty acids represent approximately 3% of the total components of *P. lentiscus* and are identified in its fruits. Djerrou et al. [29] reported that PLFO showed a good ratio (0.86) of polyunsaturated fatty acid/saturated fatty acid. The fatty acid composition consists of three dominant fatty acids: oleic acid (**138**) (monounsaturated) at 54.4%, palmitic acid (**142**) (saturated) at 22.5% and linoleic acid (**140**) (polyunsaturated) at 19.8%. The remaining percentage is relative to palmitoleic acid (**137**), gadoleic acid (**139**), linolenic acid (**141**), stearic acid (**143**) and arachidic acid (**144**).

### 2.4. Steroids (**145**–**148**)

The steroid components (**145**–**148**) were identified in PLFO extracted from *P. lentiscus* fruits (85% dark and 15% red berries), which also contains, in addition to sterols, tocopherol (**149**), carotenoids and chlorophyll [29].

However, any discrepancies in secondary metabolite concentrations may be due to the collection period as well as soil conditions.

**Figure 2:**
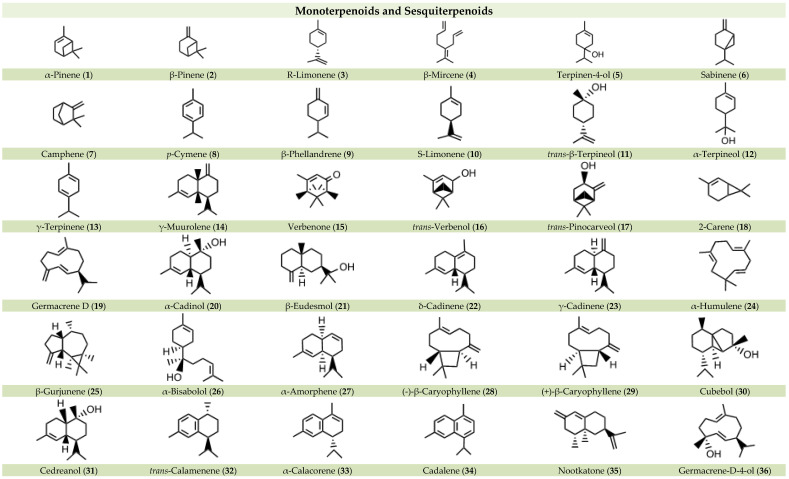

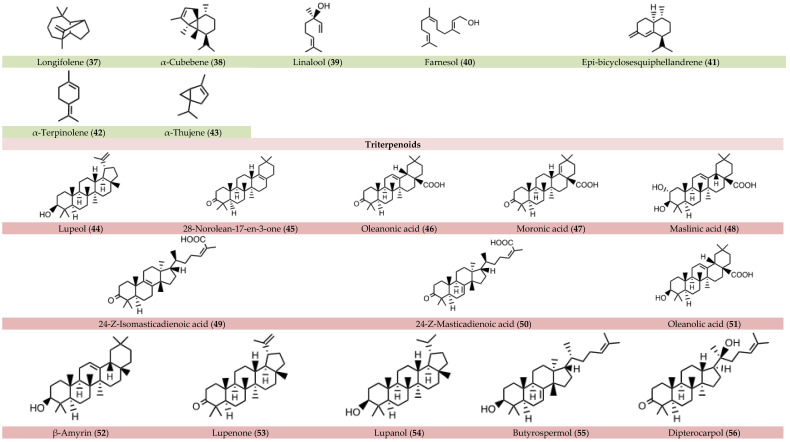

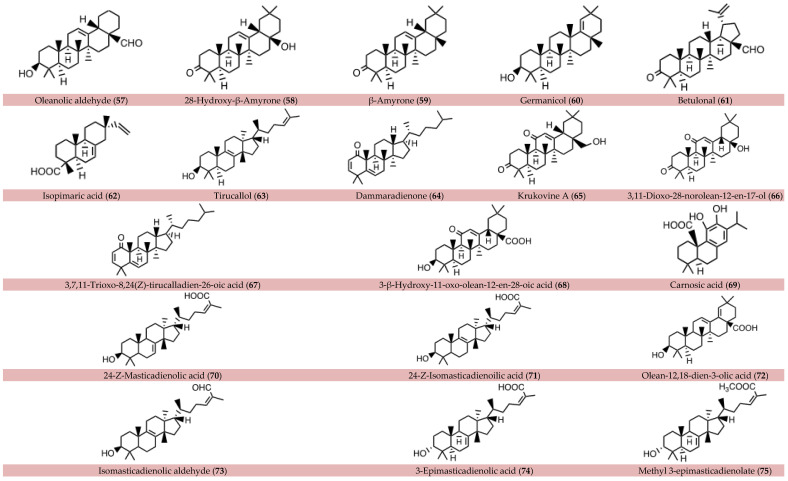

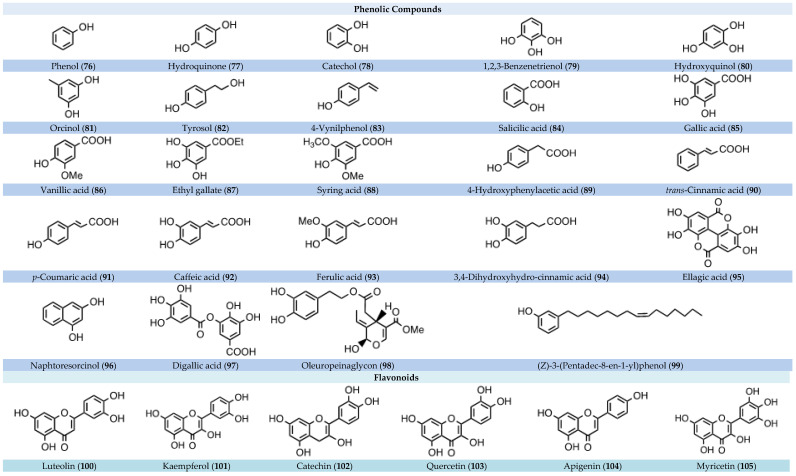

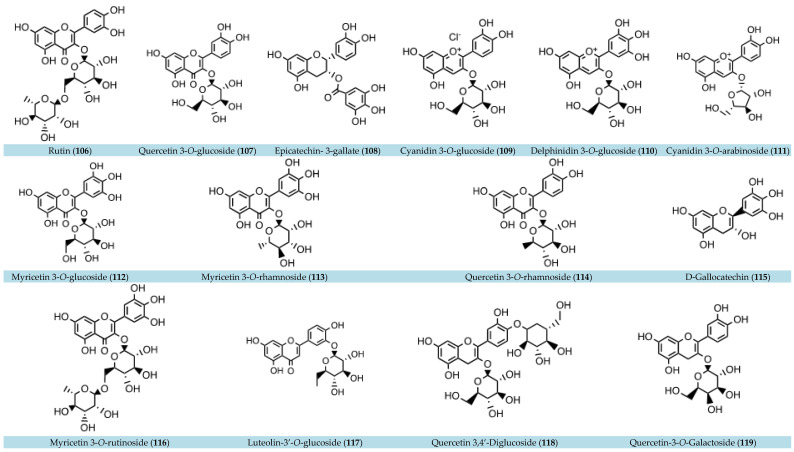

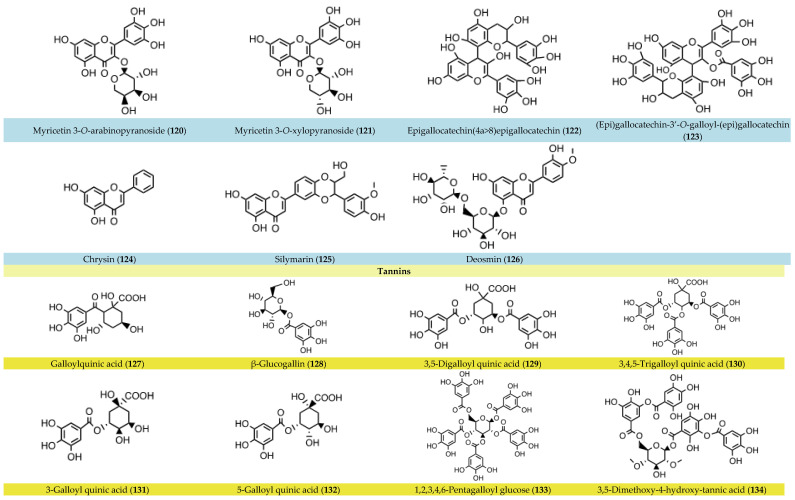

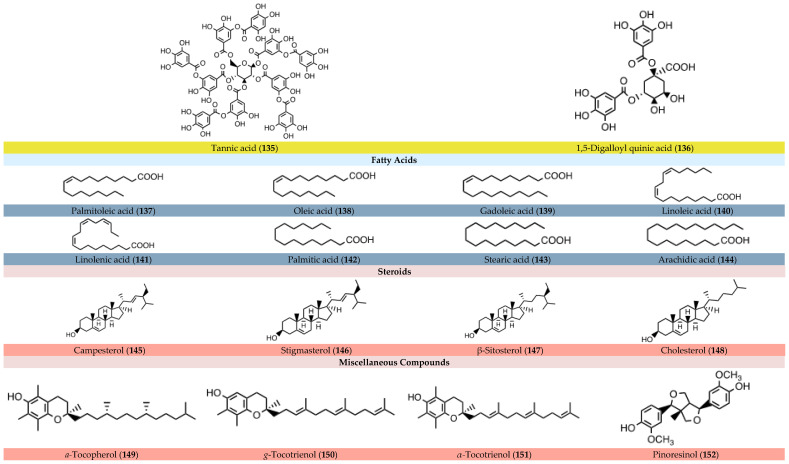

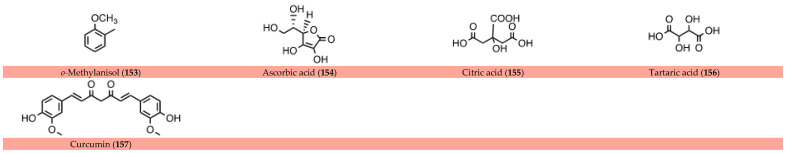
Chemical compounds identified in or isolated from *Pistacia lentiscus*.

All the compounds identified in *P. lentiscus*, divided by class of molecules, are summarized below in Figure 2.

The overall analysis of the chemical composition showed that *P. lentiscus* contains several bioactive molecules. The antidiabetic activities of extracts and/or single compounds from *P. lentiscus* are described in depth in the following section.

## 3. Antidiabetic Properties

### 3.1. In Vitro Experiments

#### 3.1.1. α-glucosidase and α-amylase Inhibition

α-glucosidase (E.C. 3.2.1.20) and α-amylase (E.C.3.2.1.1) belong to the enzyme targets in the T2D treatment. Inhibition of these enzymes results in a decrease in glucose production and absorption after a meal. In fact, these digestive enzymes first act in the mouth with the activity of the salivary α-amylase, produced by salivary glands. Then, the digestion carries on in the lumen of the small intestine with the endoglycosidase activity of the pancreatic α-amylase, producing short oligosaccharides that can be hydrolyzed by α-glucosidase. α-glucosidase, present in the brush border of the small intestine, therefore produces free glucose that can be absorbed and addressed into the blood. Thus, in diabetic patients, one strategy is to inhibit these enzymes in order to prevent postprandial hyperglycemia.

An inhibitory effect toward these enzymes by *P. lentiscus* has been reported.

Foddai et al. [22] reported the inhibition of enzymatic starch digestion by aqueous extract from *P. lentiscus* leaves and fruits. A dose-dependent reduction in glucose release from starch hydrolysis was observed, and the IC_50_ values from leaf and fruit extracts were shown to be 65.3 ± 7.4 µg/mL and 1.4 ± 0.2 µg/mL, respectively. The inhibition of α-glucosidase and α-amylase activity was also investigated separately. α-amylase inhibition exerted by ethanolic extract from leaves and fruits was reported by Mehenni et al. [24]. Both extracts showed inhibition, but it was less effective than that of acarbose, the standard inhibitor, whose leaf extract is the most active, with an IC_50_ value of 87.5 µg/mL, lower than that of the fruit extract (IC_50_ of 144.29 µg/mL).

These results were also confirmed by Tebbi et al. [21], who studied the inhibitory effect of *Pistacia lentiscus* L. black fruits against α-amylase. The fruits were extracted by choline chloride-acetic acid (ChCl-Acet) deep eutectic solvent (DES), and it was found that the extract showed a good inhibitory activity on α-amylase, with a percentage of 64.03 ± 1.21% at 25 µg/mL.

Black fruits were also evaluated by Hamdi et al. [28] for their ability to inhibit α-amylase and α-glucosidase. *Pistacia lentiscus* fatty oil (PLFO) of the extract and the unsaponifiable matter (USM) were separated and examined for the abovementioned antidiabetic activities. PLFO displayed a low inhibitory effect on α-amylase (IC_50_ > 400 μg/mL), although USM demonstrated much greater inhibitory activity, with an IC_50_ value of 180.93 μg/mL, 20 times more effective than the standard molecule, acarbose (IC_50_ of 3650.93 μg/mL). This significant inhibition of α-amylase enzyme by the unsaponifiable matter emphasizes the significance of its isolation from the oil. As regards α-glucosidase inhibitory activity, both PLFO and USM extracts exhibited good inhibitory activity (IC_50_ values of 136.47 and 155.77 μg/mL, respectively) in comparison with acarbose (IC_50_ of 275.43 μg/mL). These results indicate that the inhibition of α-glucosidase could be attributed to USM, but there was no change in the inhibitory power as a result of its separation from the oil.

According to Sehaki et al. [1], aqueous lentisk extracts from leaves, stem barks and fruits harvested from two locations in Tizi-Ouzou (i.e., littoral and mountain) presented different percentages of inhibition against α-glucosidase at a concentration of 10 µg/mL. The stem bark extracts were the most effective, with average values of 84.7 ± 5.9% (IC_50_ of 5.8 ± 0.4 μg/mL) and 69.9 ± 19.9% (IC_50_ of 7.9 ± 3.3 μg/mL) at the littoral and mountain, respectively, followed by leaves and finally the fruit extracts, which showed the lower inhibitory activity (13.6 ± 6.2%) at both locations.

Cherbal et al. [23] showed the impact of *P. lentiscus* L. hydro-methanolic leaf extract on the activities of α-amylase and sucrase enzymes. The extract reveals a good inhibition against α-amylase and sucrase activities, with IC_50_ values of 5.81 mg/mL and 9.32 mg/mL, respectively. Therefore, higher inhibition is shown by the lower IC_50_ value, indicating that the extract of *P. lentiscus* L. has more of an inhibitory effect on α-amylase than on sucrase.

An in vitro system with yeast cells suspended in a glucose solution in the presence or absence of the extract was used by the same authors [23] to examine the effect of *P. lentiscus* L. hydro-methanolic leaf extract on glucose transport across the yeast cell membrane. The process by which glucose is transported across the yeast cell membrane is also being studied as an in vitro screening technique for the hypoglycemic impact of different compounds and medicinal plants. An indicator of the amount of glucose taken up by the yeast cells is the amount of glucose that is still in the medium after a specific time. The result showed that *P. lentiscus* L. extract possesses the ability to increase glucose absorption on yeast cells in a dose-dependent manner, strongly correlated with the concentration of the extract, reaching 92.85% at 50 mg/mL.

Furthermore, essential oils and volatile molecules have recently attracted a lot of attention as potential natural substance candidates. In this context, El Omari et al. [19] examined the antidiabetic properties of the volatile components of *Pistacia lentiscus* essential oils (PLEOs), obtained from fruit extract and its main compounds, limonene and α-pinene. The results showed that PLEO, along with α-pinene (**1**) and limonene (**3**,**10**), exhibited promising antidiabetic potential, with IC_50_ values ranging from 78.03 ± 2.31 to 116.03 ± 7.42 μg/mL for α-glucosidase and from 74.39 ± 3.08 to 112.35 ± 4.92 μg/mL for α-amylase assay. In particular, limonene was found to be the most active compound, with the IC_50_ values equal to 78.03 ± 2.31 and 74.39 ± 3.08 μg/mL for α-glucosidase and α-amylase, respectively. This suggests that limonene could represent a promising target for the development of antidiabetic drugs.

Moreover, an effect of *P. lentiscus* toward α-glucosidase enzyme as a target has been reported in a cellular system [30]. In fact, this study reported the biological effects of Chios mastic extract in Caco-2 cells, a model of human small intestine mucosa. This extract, with very high contents of triterpenes, was demonstrated to modulate glucose metabolism in Caco-2 cells by reducing disaccharidase activity along with sucrase–isomaltase expression.

Overall, these studies highlight the fact that *Pistacia lentiscus* possesses the ability to inhibit crucial gastrointestinal enzymes involved in carbohydrate digestion and absorption, thus supporting its potential as an antidiabetic agent that can be useful on the management of T2D.

#### 3.1.2. Inhibition of Lipase

Pancreatic lipase (PL, EC 3.1.1.3) represents another therapeutic target for the treatment of T2D. It is the key enzyme in lipid absorption, responsible for the digestion of dietary triglycerides in the small intestine. PL catalyzes the hydrolysis of triacylglycerols into free fatty acids and monoacylglycerols, which can be absorbed by enterocytes [31]. Excessive accumulation of lipids in the pancreas may contribute to insulin-producing β-cell dysfunction, characteristic of T2D. In this context, inhibitors of pancreatic lipase are attracting much research interest due to their anti-obesity activity by delaying the lipolytic process [32]. This action would lead to a decrease in lipid absorption and thus protect the pancreas, which would restore regular insulin production from the β-cells. On this basis, Foddai et al. [22] evaluated the inhibitory effect of aqueous extracts of *Pistacia lentiscus* leaves and fruits against PL. The extracts exhibited highly significant in vitro dose-related inhibition of PL. Among them, *P. lentiscus* leaves were shown to possess an IC_50_ value of 6.1 ± 0.2 μg/mL, 20 times lower than that of *P. lentiscus* fruits (125.2 ± 12.1 μg/mL). A similar result was obtained by Cherbal et al. [33], who reported a lipase inhibition by the methanolic extract of *P. lentiscus* leaves with an IC_50_ of 2.8 μg/mL.

These results contribute to the consideration of *Pistacia lentiscus* as a potential candidate for the development of functional foods in obesity prevention and phytotherapy.

#### 3.1.3. Inhibition of 11β-Hydroxysteroid Dehydrogenase 1

11β-Hydroxysteroid dehydrogenase type 1 (11β-HSD1, E.C. 1.1.1.146) is an enzyme that is mainly expressed in the liver and adipose tissues, which catalyzes the conversion of inactive 11-ketoglucocorticoids into active 11β-hydroxy-forms. It is also known as cortisone reductase, because it is a NADPH-dependent enzyme catalyzing the reduction of inactive cortisone to active cortisol. In contrast, 11β-hydroxysteroid dehydrogenase 2 (11β-HSD2) is an NAD^+^-dependent dehydrogenase catalyzing the oxidation of cortisol to cortisone.

Since cortisol and glucocorticoids synthetized by 11β-HSD1 activity decrease glucose uptake and enhance liver gluconeogenesis, affecting carbohydrate and fat metabolism, increased enzymatic activity may result in obesity, insulin resistance and T2D [34]. Inhibition of 11β-HSD1 activity decreases insulin resistance and glucose production, representing a therapeutic target for the development of potential drugs for T2D. There are several known 11β-HSD1 inhibitors from synthetic or natural origin, the latter being mostly triterpenes. Mastic gum from *P. lentiscus* is rich in triterpenes, and the 11β-HSD1 inhibitory activity was investigated. Oleoresin, the acidic fraction and two triterpenes isolated from the acidic fraction (masticadienonic and isomasticadienonic acids) were shown to selectively inhibit 11β-HSD1 and not 11β-HSD2. The inhibitory activity was evaluated in lysed cells expressing 11β-HSD1. The IC_50_ values were reported and shown to be 1.33 and 2.10 μg/mL for oleoresin and the acidic fraction, and 2.51 and 1.94 μM for masticadienonic (**50**) and isomasticadienonic (**49**) acids, respectively. The IC_50_ value of the standard inhibitor glycyrrhetinic acid was also determined as 0.68 μM (0.32 μg/mL). This IC_50_ is slightly lower than that observed for the tested compounds, but glycyrrhetinic acid also inhibits 11β-HSD2 and is therefore not suitable for diabetes treatment. Moreover, docking analysis predicted the binding site of masticadienonic and isomasticadienonic acids and confirmed that both compounds occupied the 11β-HSD1 binding site; therefore, they inhibited the enzyme preventing the binding of its natural substrate [35].

Thus, one of the reasons why mastic gum is known for its antidiabetic effect could be represented by 11β-HSD1 inhibition and the consequent regulation of glucocorticoid metabolism.

#### 3.1.4. Biological Activity towards PPARγ

The peroxisome proliferator-activated receptors (PPARs) are nuclear fatty acid transcription factors involved in several diseases. In particular, PPARγ is the most studied member of this family; it regulates glucose and lipid pathways, with a high expression in adipose tissue and, to a lesser extent, in the liver and in the muscle. By binding to a specific ligand, PPARγ regulates the transcription of several target genes in tissues that are involved in lipid and glucose metabolism and homeostasis. PPARγ agonists, such as thiazolidinediones (TZD), act as insulin-sensitizing agents and are currently used in clinical practice for the treatment of diabetes. They normalize the glucose profile, promoting insulin-stimulated glucose uptake and suppressing hepatic gluconeogenesis [36]. Despite the beneficial effect of using such drugs, there are several undesirable side effects due to the use of TZDs, such as weight gain, fluid retention, heart failure and hepatotoxicity. The activation of PPARγ is affected by the type of agonist, which may be full or partial depending on the structural differences and activation profile. It has been reported that a partial agonist seems to induce less side effects, as observed for the full agonist TZDs [37]. In this context, the identification of PPARγ partial agonists is a promising strategy for diabetes management.

Petersen and colleagues [37] conducted a virtual screening for novel PPARγ partial agonists and identified oleanonic acid (**46**) as a potential target molecule. Oleanonic acid is a triterpene found in the oleoresin of *Pistacia lentiscus* var. Chia. Neutral and acidic fractions of oleoresin were shown to possess PPARγ activation properties. Specifically, a fraction of the acidic fraction almost exclusively contained oleanonic acid and was further characterized. Dose response analysis and a competition assay using a standard full agonist suggested that this fraction containing oleanonic acid may function as a partial agonist for PPARγ. This result was confirmed by a docking analysis of oleanonic acid, which showed the aminoacidic interaction characteristic of a partial agonist molecule.

Considering that this extract acts as partial PPARγ agonist, the activity of these subfractions of *P. lentiscus* oleoresin was less than that of a full-agonist reference compound, being about 20% of the full agonist Rosiglitazone. Otherwise, it could contribute to the activities of the gum, which have also been described towards other T2D therapeutic targets (i.e., 11β-HSD1), confirming the beneficial antidiabetic properties of the Chios mastic gum.

### 3.2. In Vivo Experiments

#### 3.2.1. Hypoglycemic Effect

##### Mice Models

To investigate the antiglycemic effect of *P. Lentiscus* on mice, diabetes is induced using two primary techniques, alloxan and streptozotocin (STZ). The alloxan-induced diabetes technique involves a single intra-peritoneal injection of a freshly prepared alloxan solution at a dose of 150 mg/kg body weight. This compound, acting as a toxic glucose analogue, selectively accumulates in pancreatic beta cells via the GLUT2 glucose transporter. Upon entry into the beta cells, alloxan undergoes a cyclic redox reaction, facilitated by intracellular thiols such as glutathione, generating reactive oxygen species (ROS) and, ultimately, hydroxyl radicals. The ensuing oxidative stress leads to the death of beta cells, resulting in insulin-dependent alloxan diabetes. Notably, alloxan also inhibits glucose-induced insulin secretion by selectively targeting the beta cell glucose sensor glucokinase [38]. On the other hand, the STZ-induced diabetes technique involves intra-peritoneal administration of STZ dissolved in a citrate buffer, typically at a dose of 60 mg/kg. Following uptake into beta cells, STZ undergoes enzymatic cleavage, separating into its glucose and methylnitrosourea components. The latter, with potent alkylating properties, induces damage to biological macromolecules, including DNA fragmentation, leading to beta cell destruction and the onset of insulin-dependent diabetes [39].

In the murine model of alloxan-induced diabetes, various experiments have explored the use of *P. lentiscus*. One study treated mice with a hydro-methanolic leaf extract at 300 mg/kg, resulting in a significant 36% reduction in blood glucose levels and an accompanying increase in plasma insulin levels [23]. Another study on crude Pistacia gum treatment at 100 mg/kg in diabetic mice showed significantly lower blood glucose levels compared to untreated diabetic mice, with crude *P. lentiscus* showing comparable effectiveness to the antidiabetic drug glibenclamide in maintaining glucose homeostasis [40]. This study also assessed the levels of liver function markers alanine transaminase (ALT) and aspartate transaminase (AST), which were significantly lowered in the *P. lentiscus*-treated group on the 21st day as compared to the diabetic untreated and glibenclamide-treated groups.

In the murine model of STZ-induced diabetes, treatment with ethanol extracts of *P. lentiscus* leaves and fruits at concentrations of 50 mg/kg and 125 mg/kg demonstrated a substantial reduction in blood glucose levels comparable to the reference drug glibenclamide. Additionally, flower extracts exhibited a milder effect, and leaf extracts restored blood glucose to normal values after a two-hour treatment, emphasizing their efficacy in diabetes management [24].

##### Human Models

Several clinical studies have demonstrated the anti-hyperglycemic effects of Chios mastic gum on human volunteers [20].

In a prospective, randomized, placebo-controlled pilot study involving 156 volunteers with total cholesterol levels > 200 mg/dL, volunteers were divided into four groups: a control group (receiving placebo), a total mastic group (receiving 1 g of total crude mastic gum capsules three times daily), a polymer-free mastic group (receiving 1 g of polymer-free mastic capsules three times daily) and a powder mastic group (receiving 2 g of crude mastic gum daily). After eight weeks, the total crude mastic gum group showed a significant reduction in fasting plasma glucose by 4.5 mg/dl (*p* < 0.05), particularly in overweight and obese individuals with body mass index (BMI)  > 25. The other groups did not exhibit significant alterations in glucose levels, although there was a trend towards reduction. No adverse events were reported, and there were no changes in BMI, liver enzymes or renal function markers [20].

In another double-blind, placebo-controlled, randomized trial involving healthy Japanese men over 40 years old, Chios mastic gum intake alone and in combination with physical activity led to significant reductions in insulin levels and insulin resistance compared to a control group. Specifically, CMG intake alone reduced insulin resistance values at 6 months. Moreover, CMG intake combined with exercise reduced insulin resistance already at 3 months, suggesting its importance, along with physical activity, in effectively managing glucose metabolism [41].

#### 3.2.2. Hypolipidemic Effect

Several studies have investigated the hypolipidemic effects of *Pistacia lentiscus*, both in animal models and human subjects, highlighting its potential therapeutic value in managing lipid-related disorders.

##### Mice Models

In mice with induced hypercholesterolemia by administering food containing high cholesterol (1%) for thirty days, administration of aqueous leaf extract of *P. lentiscus* resulted in significant reductions in total cholesterol (from 253 ± 31.60 mg/dL to 154.6 ± 18.10 mg/dL), triglycerides (from 200 ± 12.32 mg/dL to 97.6 ± 3.57 mg/dL) and LDL-cholesterol levels (from 160 ± 31.60 to 145.88 ± 9.76 mg/dL). Similarly, treatment with ethanolic leaf extract significantly decreased total cholesterol (154.6 ± 18.10 mg/dL), triglyceride (71.2 ± 4.38 mg/dL) and LDL-cholesterol (99.36 ± 18.77 mg/dL) levels compared to the hypercholesterolemic control group, indicating its potential as a hypolipidemic agent [42]. In STZ-induced diabetic mice, low and high doses of Chios mastic gum resulted in improvements in lipid profiles. After 4 weeks of CMG administration, serum triglyceride levels were significantly reduced in both the low-dose- and high-dose-treated groups compared to the control group. Additionally, at the end of the 8-week study period, the low-dose-treated group exhibited further improvements in serum total cholesterol, LDL and triglyceride levels, along with increased HDL levels, compared to the control group [43].

##### Rabbit Models

In rabbit models, mastic total extract without polymer and its neutral fraction demonstrated significant reductions in myocardial infarction size and atherosclerosis, alongside notable hypolipidemic effects in hypercholesterolemic rabbits. Additionally, histological analysis revealed a reduction in subintimal lipid accumulation and foamy macrophages in aortic tissue samples from rabbits treated with both extracts, suggesting anti-atherosclerotic and anti-lipidemic properties [44]. A chemical characterization of these samples was performed, and the identified compounds are reported in Table 1.

Likewise, in rabbits fed with a hyperlipidemic diet containing egg yolk, *P. lentiscus* fatty oil was administered. The results showed that egg yolk consumption significantly elevated total cholesterol, triglycerides and LDL/HDL ratio compared to the control group. However, treatment with *P. lentiscus* fatty oil or simvastatin significantly reduced total cholesterol, triglycerides and LDL. Specifically, triglyceride levels decreased by 50.75% compared to the egg yolk consumption group without fatty oil. No significant changes were observed in HDL levels [29]. Fatty acid composition has been determined, and the results are reported in Table 1.

##### Human Models

CMG has been extensively studied in several randomized controlled trials to assess its effects on lipid and glucose metabolism, as well as its potential therapeutic benefits in managing various metabolic disorders. In the study described in the previous section (Section 3.2.1) involving healthy Japanese men over 40 years old, CMG intake alone and in combination with physical activity led to significant reductions in serum triglycerides and helped manage glucose metabolism [41].

Another in vivo human study focused on Chios Mastiha Essential Oil (CMEO) demonstrated significant improvements in blood lipid profile, including reductions in triglycerides and LDL, along with other metabolic markers in individuals with abdominal obesity and metabolic abnormalities. Moreover, a decrease in systolic blood pressure and ALT levels was found only after CMEO intake, and a reduction in weight, percentage of body fat and visceral fat and also an improvement in health-related quality-of-life scores, both physical and mental, were found in the CMEO group compared to controls [45].

Furthermore, total mastic gum also showed the ability to reduce total cholesterol levels by 11.5 mg/dL (*p* < 0.05), with a stronger effect observed in overweight and obese individuals [20].

Finally, 133 participants aged over 50 were randomly assigned to either a high-dose group consuming 5 g of mastic powder daily or a low-dose group receiving a Chios mastic solution. Throughout the study duration, the high-dose group exhibited significant reductions in total cholesterol, LDL, total cholesterol/HDL ratio, lipoprotein, apolipoprotein A-1, apolipoprotein B, SGOT, SGPT and gamma-GT levels. However, glucose, HDL and triglyceride levels remained unchanged in this group. Interestingly, only male subjects in the low-dose group displayed a notable decrease in serum glucose levels. These findings suggest potential benefits of Chios mastic powder in improving lipid profiles and hepatic enzymes [46]. Further research is necessary to fully elucidate these effects and their clinical implications.

All the antidiabetic activities of *Pistacia lentiscus* extracts or isolated compounds are reported in Table 1, together with identified compounds for each active extract, where reported in the literature.

**Table 1:** Biological activities of extracts or single compounds of *Pistacia lentiscus* and chemical composition identified in different parts of the plant.

Antidiabetic Activity						
Plant Part	Origin	Type of Extract/ Active Compounds	Compound	Results		Ref.
Leaves	Algeria	Methanolic	Gallic acid (**85**) Vanillic acid (**86**) *p*-Coumaric acid (**91**) Caffeic acid (**92**)	Inhibition of pancreatic lipase (IC_50_ = 2.8 μg/mL) Inhibition of α-amylase (IC_50_ = 5.81 mg/mL) and sucrase (IC_50_ = 9.32 mg/mL). Increase in glucose transport across the yeast cell membrane	In vitro	[23,33]
				Hypoglycemic effect: reduction in blood glucose level and increase in insulin level	In vivo (Rat)	
Leaves	Algeria	Aqueous and ethanolic	n.r.	Hypolipidemic effect: reduction in triglycerides, total cholesterol and LDL-cholesterol levels	In vivo (Mice)	[42]
Leaves	Italy (Sardinia)	Aqueous	Myricetin (**105**) Quercetin-3-*O*-rutinoside (rutin) (**106**) Quercetin 3-*O*-glucoside (**107**) Myricetin 3-*O*-rhamnoside (**113**) Myricetin 3-*O*-rutinoside (**116**) Quercetin-3,4’-diglucoside (**118**) Quercetin-3-*O*-galactoside (**119**) Myricetin-3-*O*-arabinopyranoside (**120**) Myricetin-3-*O*-xylopyranoside (**121**)	Decreases in enzymatic starch hydrolysis (IC_50_ = 65.3 μg/mL for leaf and IC_50_ = 1.4 mg/mL for fruit extract) Inhibition of pancreatic lipase (IC_50_ = 6.1 μg/mL for leaf and IC_50_ = 230.7 μg/mL for fruit extract)	In vitro	[22]
Fruits			Gallic acid (**85**) Quercetin-3-*O*-rutinoside (rutin) (**106**) Quercetin 3-*O*-glucoside (**107**) Myricetin 3-*O*-glucoside (**112**) Luteolin-3’-*O*-glucoside (**117**) 3,5-Digalloyl quinic acid (**129**) 3-Galloyl quinic acid (**131**) 5-Galloyl quinic acid (**132**) 1,5-Digalloyl quinic acid (**136**)			
Fruits	Morocco	Essential oils	α-Pinene (**1**) (R) Limonene (**3**) (S) Limonene (**10**)	Inhibition of α-amylase (IC_50_ = 112.35 μg/mL for essential oils, IC_50_ = 82.12 μg/mL for α-pinene and IC_50_ = 74.39 μg/mL for limonene) Inhibition of α-glucosidase (IC_50_ = 116.03 μg/mL for essential oils, IC_50_ = 95.62 μg/mL for α-pinene and IC_50_ = 78.03 μg/mL for limonene)	In vitro	[19]
Leaves Fruits	Algeria	Ethanolic	Salicylic acid (**84**) Gallic acid (**85**) Syringic acid (**88**) 3,4-Dihydroxyhydro-cinnamic acid (**94**) Ellagic acid (**95**) Luteolin (**100**) Catechin (**102**) Quercetin 3-*O*-rhamnoside (**114**)	Inhibition of α-amylase (IC_50_ = 87.5 μg/mL for leaf and IC_50_ = 144.29 μg/mL for fruit extract).	In vitro	[24]
				Hypoglycemic effect: reduction in blood glucose level	In vivo (Mice)	
Leaves Stem barks Fruits	Algeria	Methanolic	Epigallocatechin(4a > 8)epigallocatechin (**122**) 3,5-Digalloyl quinic acid (**129**) (Epi)gallocatechin-3-*O*-galloyl-(Epi)gallocatechin (**123**) β-Glucogallin (**128**)	Inhibition of α-glucosidase (IC_50_ = 5.8 μg/mL for littoral and IC_50_ = 7.9 μg/mL for mountain stem bark extract)	In vitro	[1,2]
Black fruits	Algeria	Deep Eutectic Solvent (DES)	Catechol (**78**) Gallic acid (**85**) Cinnamic acid (**90**) Coumaric acid (**91**) Caffeic acid (**92**) Kaempferol (**101**) Catechin (**102**) Quercetin (**103**) Rutin (**106**) Cyanidin-3-*O*-glucoside (**109**) Chrysin (**124**) Silymarin (**125**) Deosmin (**126**) Ascorbic acid (**154**) Citric acid (**155**) Tartaric acid (**156**) Curcumin (**157**) 3,5-Dimethoxy-4-hydroxy-tannic acid (**134**) Tannic acid (**135**)	Inhibition of α-amylase	In vitro	[21]
Black fruits	Algeria	Fatty oil (PLFO) and unsaponifiable matter (USM)	n.r.	Inhibition of α-amylase (IC_50_ > 400 μg/mL for PLFO and IC_50_ = 180.93 μg/mL for USM) Inhibition of α-glucosidase (IC_50_ = 163.47 μg/mL for PLFO and IC_50_ = 155.77 μg/mL for USM)	In vitro	[28]
Chios mastic gum	n.r.	Oleoresin and its neutral and acidic fraction/oleanoic acid	Oleanoic acid (**51**)	PPARγ agonists	In vitro Virtual screening	[37]
Chios mastic gum	n.r.	Oleoresin and its acidic fraction/masticadienonic and isomasticadienonic acid	28-Norolean-17-en-3-one (**45**) Isomasticadienonic acid (**49**) Masticadienonic acid (**50**) Oleanolic acid (**51**) Masticadienolic acid (**70**) 3-Epimasticadienolic acid (**74**) Methyl 3-epimasticadienolate (**75**)	Inhibition of 11*β*-HSD1 (IC_50_ = 1.33 μg/mL for oleoresin, IC_50_ = 2.10 μg/mL for acidic fraction, IC_50_ = 2.51 μg/mL for masticadienonic acid IC_50_ = 1.94 μg/mL for isomasticadienonic acid)	In vitro Virtual screening	[35]
Chios mastic gum	Italia	Supermastic	Oleanonic acid (**46**) Moronic acid (**47**) Maslinic acid (**48**) Isomasticadienonic acid (**49**) Masticadienonic acid (**50**) Oleanolic acid (**51**) Masticadienolic acid (**70**) Isomasticadienolic acid (**71**)	Inhibition of disaccharidase activity in Caco-2 cells Reduction in sucrase-isomaltase expression	In vitro	[30]
Chios mastic gum	Pakistan	Crude gum powder	n.r.	Hypoglycemic effect: reduction in blood glucose level	In vivo (Rat)	[40]
Chios mastic gum	Greece	Crude gum	n.r.	Hypoglycemic and hypolipidemic effect: reduction in blood glucose level and reduction in serum triglyceride, total cholesterol and LDL levels	In vivo (Mice)	[43]
Chios mastic gum	Greece	Total mastic extract without polymer	28-Norolean-17-en-3-one (**45**) Oleanoic acid (**46**) Moronic acid (**47**) 24Z-Isomasticadienoic acid (**49**) 24Z-Masticadienoic acid (**50**) Olean-12,18-dien-3-olic acid (**72**)	Hypolipidemic effect: reduction in total cholesterol and LDL circulatory levels	In vivo (Rabbit)	[44]
		Neutral mastic fraction	Butyrospermol (**55**) Oleanolic aldehyde (**57**) β-Amyrone (**59**) Betulonal (**61**) Tirucallol (**63**) Dammaradienone (**64**)			
Fruits	Algeria	Fatty oil	Oleic acid (**138**) Linoleic acid (**140**) Palmitic acid (**142**)	Anti-hyperlipidemic effect: reduction in total cholesterol, LDL-cholesterol and triglyceride levels	In vivo (Rabbit)	[29]
Chios mastic gum	Greece	Crude gum powder	n.r.	Hypoglycemic and hypolipidemic effect: reduction in blood glucose and total cholesterol level	In vivo (Human)	[20]
Chios mastic gum	Japan	Capsules of gum powder	Camphene (**7**) Verbenone (**15**) Linalool (**39**) α-Terpinolene (**42**) 28-Norolean-17-en-3-one (**45**) Moronic acid (**47**) Masticadienonic acid (**50**) Oleanolic acid (**51**) Oleanolic aldehyde (**57**) β-Amyrone (**59**) Tirucallol (**63**) Dammaradienone (**64**) Masticadienolic acid (**70**) Isomasticadienolic acid (**71**) Isomasticadienolic aldehyde (**73**)	Hypoglycemic and hypolipidemic effect: reduction in insuline resistence and reduction in serum triglycerides	In vivo (Human)	[41]
Chios mastic gum	n.r.	Mastic solution	n.r.	Hypoglycemic and hypolipidemic effect: reduction in blood glucose level and total cholesterol, LDL, total cholesterol/HDL ratio	In vivo (Human)	[46]
Chios mastic gum	n.r.	Essential oils in gel capsules	α-Pinene (**1**) β-Pinene (**2**) (R) Limonene (**3**) β-Mircene (**4**) Camphene (**7**) (−) β-Caryophyllene (**28**) (+) β-Caryophyllene (**29**) α*-*Thujene (**43**) *o*-Methylanisol (**153**)	Hypolipidemic and anti-obesity effect: reduction in triglycerides and LDL	In vivo (Human)	[45]

n.r.: data not reported in literature.

## 4. Conclusions and Future Directions

The present review highlighted the health-promoting activities of *P. lentiscus* as a promising source of bioactive compounds that could be useful for the development of novel type 2 diabetes treatments. *P. lentiscus* was revealed to possess a significant importance in conventional medicine, also supported by the European Medicines Agency, which has officially recognized *P. lentiscus* as an herbal medicinal product. Here, we have presented all the available information concerning its chemical composition and antidiabetic properties. In fact, several extracts and compounds from different parts of the plant showed activity against one or more targets implicated in T2D treatment. Unlike T1D, T2D is a complex pathology that needs a combination therapy using two or more drugs against totally different targets in order to reduce hyperglycemia and hyperlipidemia, both contributing to diabetic disease. Though combination therapy can be effective, it often exacerbates side effects. The ultimate objective in T2D management is to identify a single drug capable of acting on multiple targets to mitigate the complications associated with polypharmacy. As reported from the literature, *P. lentiscus* showed in vitro (in cell-free and in cellular systems) and in vivo activities towards more targets, emerging as a good candidate for multitarget drug discovery in T2D treatment. A limitation of the data we collected is that each single article focused on the activity of an extract/part of plant toward one or two specific targets. Thanks to the overview of all activities for each extract provided by this review, future research could aim to identify a single extract with simultaneous multiple activities toward different targets.

Additionally, we have compiled a summary of the compounds found in *P. lentiscus* and the extracts in which each compound has been identified. Since the extracts tested are multi-component mixtures, and their biological activities were presumably derived from a synergic effect, all the active compounds have not been yet identified. Thus, from the analysis of all the data present in the literature, one future perspective could be to identify which compounds are responsible for the activities of the extracts, with the possibility of identifying novel and potent multitarget active molecules.
